# A one-step chemical treatment to directly isolate microcrystalline cellulose from lignocellulose source

**DOI:** 10.1186/s40643-025-00920-6

**Published:** 2025-08-22

**Authors:** Thai Anh Do, Van Quyen Nguyen, Thi Minh Chau Nguyen, Thi Hang Nga Nguyen, Thi Huong Le

**Affiliations:** 1 Department of Advanced Materials Science and Nanotechnology, University of Science and Technology of Hanoi, Vietnam Academy of Science and Technology, 18 Hoang Quoc Viet, Cau Giay, Hanoi, 11307 Vietnam; 2 Faculty of Chemistry, University of Science, Vietnam National University, 19 Le Thanh Tong, Hoan Kiem, Hanoi, Vietnam; 3 Faculty of Mechanical Engineering, Thuy Loi University, 175 Tay Son, Dong Da, Hanoi, Vietnam

**Keywords:** Lignocellulosic source, Microcrystalline cellulose, Peracetic acid, Ionic cellulose conductor

## Abstract

In the study, we report an effective and one-step chemical treatment for directly isolating microcrystalline cellulose (MCC) with a width of 10–50 μm and length of 100–1000 μm from various agricultural waste sources. This chemical treatment uses a peracetic acid solution combined with 2% H₂SO₄ at 80 °C for 2 h. After this one -step chemical treatment, we observed significant changes in chemical composition. For example, the cellulose percentage substantially increased across all biomass sources: from 26.73 to 55.94% for dragon fruit foliage, and from 41.26 to 64.13% for corn cob, and from 17.86 to 67.38% for banana pseudostem. Simultaneously, the lignin content decreased from 22.05 to 1.03%, 12.74 to 3.76% and 14.27 to 2.73%, respectively for dragon fruit foliage, corn cob, and banana pseudostem. XRD confirmed that the crystallinity of the bleached cellulose is high (above 65%), and elemental analysis showed the existence of only O and C elements in the bleached sample. We attributed the effectivity of our chemical treatment to the combination effect of solvent polarity, strong oxidative agent and H_2_SO_4_ acid, which allows the removal of extractive, the lignin and partially hydrolysis hemicellulose component simultaneously at the short time. Our microcrystalline cellulose can serve as an effective matrix for gel polymer electrolytes, exhibiting good mechanical behavior and excellent electrical properties, with highest ionic conductivity of 197 mS. cm^−1^. Our study opens a new, simple path to directly isolate microcrystalline cellulose from lignocellulosic source and extend the application of microcrystalline cellulose toward the field in energy storage and conversion.

**Graphical abstract:**

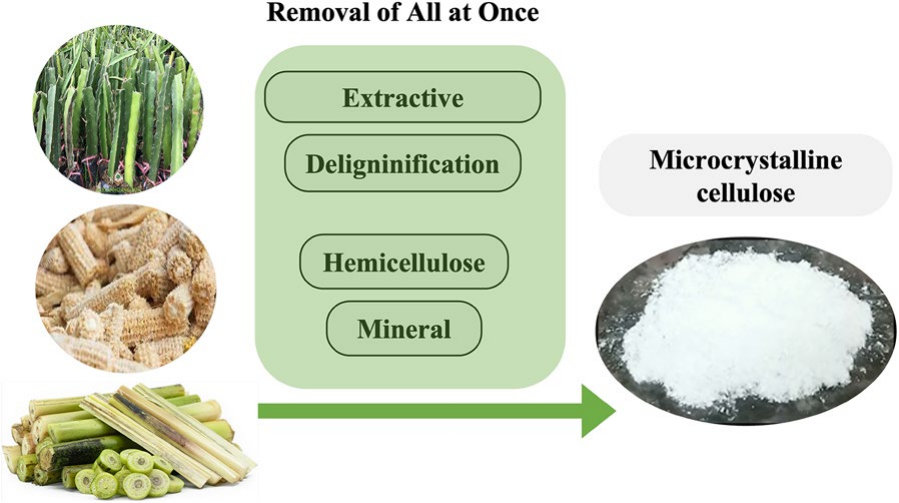

**Supplementary Information:**

The online version contains supplementary material available at 10.1186/s40643-025-00920-6.

## Introduction

Microcrystalline and nanocrystalline cellulose have been considered some of the most promising building blocks due to their abundance in nature, natural regeneration in the biosphere, and unique physicochemical properties, including natural degradability, environmental friendliness, and biocompatibility (Moon et al. 2011; Heise et al. 2021; Kontturi et al. 2018; Norrrahim et al. 2021; Klemm et al. 2011; Chen et al. 2024; Gao et al. 2024; Dong et al. 2024). Microcrystalline and nanocrystalline cellulose are recently emerged as potential materials for the application in different fields including food, (Debnath et al. 2024; Homyuen et al. 2023) cosmetic and biomedical, (Cabua et al. 2024; Basagaoglu Demirekin et al. 2015; Kumar and Chauhan 2022; Bai et al. 2018) electrical, energy and environmental sector (Aggarwal et al. 2023; Ong et al. 2022). For examples, Paola et al. reported a general protocol to produce antimicrobial materials based on cellulose and peptides. They found that the cellulose conjugated to the peptides lasioglossin-III and TBKKG6A showed a significant reduction in the concentration of viable cells compared to unmodified cellulose (Sperandeo et al. 2020). Jincheng Miao et al. reported the novel cationic adsorbent based on MCC functionalized with 2,3-epoxypropyltrimethylammonium chloride which improved the removal efficiency up to 99% for acid yellow 128 (Miao et al. 2023). Jiaxiang Zhao et al. reported that a microcrystalline cellulose-based elastomer displayed an impressive erasure effect combined with good biodegradability and they attributed to its unique microstructure with a very high loading of MCC (75 wt %) (Zhao et al. 2024). Chunpeng Yang et al. reported a simple and effective strategy to convert non-conductive cellulose into high ionic conductivity cellulose through the coordination of copper ion (Cu^2+^) within cellulose nanofibrils, which show rapid transport of Li + ions along the cellulose chains (up to 1.5 × 10^–3^ S.cm^−1^) (Yang et al. 2021). Qi Dong et al. recently reported the same approach for the scalable and cost-effective synthesis of an ionic-conducting (e.g., Na +) cellulose derived polymer with high ionic conductivity (e.g., 0.23 S.cm^−1^ in 20 wt% NaOH at 25 °C) (Dong et al. 2022).

**Scheme 1:**
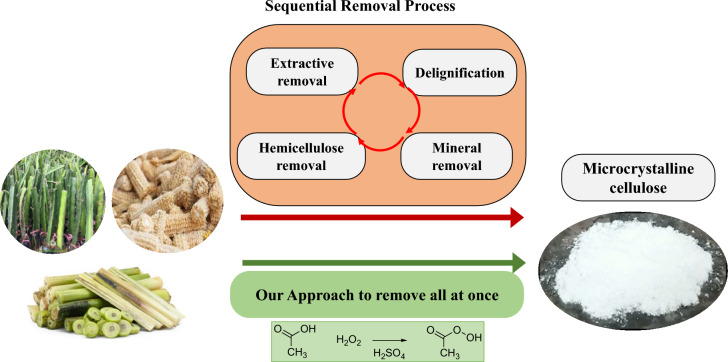
Schematic presents general procedures to extract microcrystalline cellulose (MCC) from lignocellulosic source. Red arrow describes a traditional method, which remove all the chemical components in a sequence, as illustrated as the red semi-circle arrow. Green arrow describes our approach based on peracetic acid, that can remove all the chemical components at once

Among several crystalline cellulosic derivatives, microcrystalline cellulose (MCC) is the most common form. It typically manifests as a particles with a diameter of around50 µm and a length of 100–1000 µm. Generally, microcrystalline cellulose (MCC) is produced from lignocellulose source, especially agricultural waste, by several chemical process as illustrated in Scheme 1. To extract MCC, other chemical components of the lignocellulosic source such as waxes, extractives, lignin, hemicellulose, minerals components must be removed. Generally, this process, known as the bleaching process, can be divided into three main steps. The first step is to remove waxes and extractives using organic solvent. The second step involve removing the lignin component using strong oxidative agents such as NaOCl, H_2_O_2_ or NaClO_2_ in an alkaline environment. This delignification process breaks down the complex lignin structure into smaller, soluble fragments that can be washed away. The last step is to remove minerals and partially hydrolyze amorphous regions to obtain microcrystalline cellulose with a crystallinity ranging from 55 to 80% (Haldar and Purkait 2020). This bleaching process strongly depends on the content of the chemical components, in other word, the lignocellulosic source. It is often repeated several times to obtain the MCC, which can lead to serious problems such as time and energy consumption, equipment corrosion, and severe environmental pollutions (Haldar and Purkait 2020).

To overcome these drawbacks, two main approaches have been developed in recent years. The first approach involves adding mechanical treatment during the bleaching process. Mechanical pretreatment methods such as ball milling, ultrasonication, and high-pressure homogenization (Lee et al. 2018; Nuruddin et al. 2016; Chowdhury et al. 2019) can also reduce the need for harsh chemicals by physically breaking down the biomass structure. These mechanical methods increase the surface area and accessibility of the cellulose fibers, thereby improving the efficiency of subsequent chemical treatments. Steam explosion (Hongzhang and Liying 2007) has also emerged as an effective pretreatment method. Steam explosion uses high-pressure steam followed by rapid decompression to break down the lignocellulosic structure. This approach still requires another chemical treatment to eventually obtain MCC. The second approach is to develop an effective chemical treatment that removes all the chemical components. For examples, enzyme treatment (Yuan et al. 2022; Lou et al. 2022) methods can selectively degrade specific components of the lignocellulosic biomass. Enzymatic treatments offer several advantages including high specificity, mild reaction conditions (moderate temperature and pH), and minimal environmental impact. Organic acid hydrolysis such as with formic acid (Zhang et al. 2017) and citric acid (Hindi 2024), has also shown effective delignification while being less corrosive than mineral acids. Ionic liquid treatment using 1-allyl-3-methylimidazolium chloride ([AMIM]Cl) (Wang et al. 2011) or 1-ethyl-3-methylimidazolium acetate ([EMIM]Ac), (Sun et al. 2013) are also able to break down the hydrogen bonding network in lignocellulosic materials, leading to efficient separation of cellulose from lignin and hemicellulose. Deep eutectic solvent (DES) particularly choline chloride/urea (Xu et al. 2023) and choline chloride/lactic acid (Soleimanzadeh et al. 2023) can fractionate lignocellulosic biomass components under mild conditions, exhibiting high selectivity and recyclability. Recently, Li et al. reported an effective approach using peroxyformic acid at a mild temperature of 50 °C for 12 h to delignify natural bamboo to extract high strength cellulose macrofibers (Li et al. 2021). In their study, they suggested that the solution quickly diffuses throughout the material, allowing the hydroxyl (HO•) and superoxide anion (O2• −) radicals generated from the peroxyformic acid to attack the electron-rich aromatic rings and olefinic side-chain structures of lignin. The oxidative cleavage of the lignin side chains, such as those in the β-β (resinol), β-5 (phenylcoumaran) and β-1 (spirodienone) structures, as well as oxidative ring opening, further breaks down lignin into smaller molecular fragments. These approaches are promising candidates because they can be recyclable, and have low corrosive power. Especially they are fully accord with the 12 principles of green chemistry and have emerged as a promising new generation of green solvent (Sharma et al. 2020; Hu et al. 2022).

Recently, peracetic acid (PAA), an organic peroxide with strong oxidative activity, has gained increasing attention in biomass processing. PAA pretreatment offers strong oxidizing properties and operates effectively under mild reaction conditions, making it a suitable approach aligned with green chemistry principles and sustainable development. Studies have demonstrated that peracetic acid can significantly enhance lignin and hemicellulose removal while preserving the cellulose fraction, thereby improving enzymatic saccharification (Barbash et al. 2023a, 2024; Qin et al. 2018; Golbaghi et al. 2017; Pascoli et al. 2022; Uju et al. 2016; Tian et al. 2021; Lyu et al. 2021). For example, Lyu et al. reported the development of a one-step pretreatment method—PAM (peracetic acid combined with maleic acid)—for lignocellulose fractionation of corn stover. Under optimized conditions (1.5 wt% PAA and 3 wt% maleic acid at 130 °C for 1 h), the process effectively removed 88.21% of hemicellulose and 87.77% of lignin, while retaining 86.83% of cellulose in the solid residue, indicating efficient component separation. However, the study did not evaluate or demonstrate the ability of the PAM process to fractionate or disintegrate cellulose fibers (Lyu et al. 2021). Barbash et al. reported the extraction of cellulose from corn crop residues using a solution of peracetic acid, however this process required two more steps with 5% NaOH treatment at ~ 100 °C for 2 h (Barbash et al. 2023b). Similarly, a PHP system combining phosphoric acid and hydrogen peroxide (with 50–80 wt% phosphoric acid and 1.8–12.4% hydrogen peroxide) facilitated in-situ generation of PAA, achieving over 83% delignification and 87% cellulose retention in wheat straw, suggesting that acetyl groups released from hemicellulose and lignin degradation can be leveraged for self-generation of PAA under acidic conditions (Tian et al. 2021).

In the present work, we demonstrate a single on-step method to directly extract microcrystalline cellulose using a mixture of PAA solution, as illustrated a green arrow in Scheme 1. We applied this method to several lignocellulosic sources collected from agricultural waste, including dragon fruit foliage, banana pseudostem and corn cob, as the starting material. The chemical components of lignocellulosic source and the bleached cellulose were quantified and compared, the physicochemical properties of all the samples were characterized by several physical techniques. We observed that the MCC can be directly obtained from the bleaching of the lignocellulose with the peracetic solution with 2% H_2_SO_4_ in a single chemical reaction treatment at 80 °C for 2 h, and the solution can be reused for at least 3 times. The structure of microcrystalline cellulose remained similar before and after the bleaching process. We also observed that MCC obtained from this study can serves as an effective matrix for gel polymer ion-conducting electrolytes, demonstrating promising mechanical and electrical properties with a high ionic conductivity of approximately 0.2 S.cm^−1^.

### Experimental section

**Chemicals**: Fine-dried Dragon Fruit foliage (DFF), corn cob (CC) and banana pseudostem (BP) were obtained by mechanically milling air-dried samples, it was then stored in a sealed container. Analytical grade chemicals including hydrogen peroxide, acetic acid and sulfuric acid were purchased from Sigma-Aldrich and used without further purification.

**Extraction of Microcrystalline cellulose**: The chemical solution was prepared by mixing hydrogen peroxide (30%) and acetic acid glacial in equal volumes (20 mL of H_2_O_2_ and 20 mL of acetic acid glacial), then added 2% (w/w) sulfuric acid 98% (~ 0.45 mL). The biomass to liquor ratio was 1:10 (g/mL), and the reaction was conducted at a temperature of 80 °C degrees for a duration of 2 h. The reaction was then terminated by adding cold water, the solid residue was washed and filtered by distilled water until neutralization.

### Chemical composition analysis

To determine the chemical content of raw biomass and bleached samples, we used the protocol published by national renewable energy laboratory with some modification (Sluiter et al. 2008; Wychen and Laurens 2013). Particularly, for extractive content, the procedure was conducted: 0.3 g of biomass sample was weighed and placed in a beaker. Then, 30 mL of ethanol was added to the beaker, and the mixture was subjected to an oil bath at 70 °C for a duration of 3 h. Subsequently, the solid residue was filtered and washed with hot ethanol ten times, using 2 mL of ethanol each time. The residue was then dried until a constant weight was achieved and cooled to room temperature. The extractive content was calculated as the weight loss observed after the treatment.

*For lignin content*: A extractive-free sample weighing 0.3 g was placed in a beaker. To the beaker, 3 mL of 72% sulfuric acid was added, and the mixture was kept in a water bath at 30 °C for 1 h. After the hour elapsed, 84 mL of distilled water was added to the beaker. The mixture was then subjected to autoclaving at 120 °C for 1 h. Subsequently, the solid residue was filtered and washed with hot distilled water to remove any excess acid. The acid insoluble residue was dried at 105 °C until a constant weight was achieved, cooled in a desiccator to room temperature, and weighed as m_1_. The residue was then placed into a muffle furnace at 575 °C until a constant weight was obtained. After cooling in a desiccator to room temperature, the weight of the resulting ash was recorded as m_2_. The acid insoluble lignin content was calculated by subtracting m_2_ from m_1_. The amount of acid soluble lignin was determined using UV–Vis Spectroscopy.

*For hemicellulose content*: A weigh 0.3 g of extractive free sample added to 3 mL of 0.5 M NaOH in a beaker placed in an oil bath at 80 °C for 3.5 h. After that, the residue is washed with distilled water to neutral pH then it is dried to constant weight, cooled to room temperature. The amount of hemicellulose content is the weight loss after the treatment.

*For ash content*: A weigh 0.1 g of biomass sample into a pre-weighed crucible, ignite the sample until no more smoke or flame appears, followed by dry oxidation in the 575 °C muffle furnace for 5-6 h. The crucible and sample are then cooled to room temperature in a desiccator. Record the weight of ash content.

**Preparation of Ionic gel Conductor**: The dissolution of microcrystalline cellulose was performed based on the previous report (Budtova and Navard 2016b; Ogawa et al. 2014a; Qi et al. 2011). First, the sodium cuprate solution was prepared by adding dropwise 10 mL of 0.01 M CuSO_4_ solution into 10 mL of 6.25 M NaOH solution. The solution was then cooled in a refrigerator at − 12 °C for 20 min as reported previously (Qi et al. 2011). Subsequently, 4% (w/w) of cellulose samples were added to the solution and mixed thoroughly at ambient temperature for 2 min. After that the solution was kept at − 12 °C for 20 min again. The dissolution process, including mixed at ambient temperature and cooled it at − 12 °C, was carried out until the solution becomes clear.

The gelation process was then initiated by adding 13% (v/v) epichlorohydrin (ECH) and stirring vigorously for 20 min at ambient condition as reported previously (Zhao et al. 2016). After that, the solution was poured into three cylindrical molds with diameter of 16 mm and varying thicknesses (1 mm, 3 mm, and 10 mm). The mold with the pre-hydrogel solution was incubated at 4 °C. The crosslinked hydrogels were obtained from the mold carefully and used for electrical measurement.

### Materials characterization

The chemical structure of the samples was determined by Fourier transform infrared (FT/IR 4700, JASCO, JAPAN) with attenuated total reflectance (ATR) mode at a wavenumber range of 400–4000 cm^−1^ with a step of 2 cm^−1^ and for 32 scans.

Field Emission Scanning Electron Microscopy (FE-SEM). The microcrystalline cellulose solution was spin coated on SiO2 (100) wafer, and then the wafer sample was dried at 40 °C under vacuum overnight. The sample was then coated with 15 nm of Ti by e-beam evaporator (lesker PVD-75) at vacuum 10^–6^ torr and depositing rate 0.2 nm.s^−1^. The sample was then imaged by FE-SEM (JEOL, JSM-IT800) at few kV. EDS mode was conditioned by using an accelerating voltage of 20 kV, a working distance of 10 mm, and the accumulation time of 1 min.

The crystallinity of each sample was determined by X-ray diffraction (D8- ADVANCE X-ray powder diffractometer, Bruker, Germany), operating at 40 kV voltage and 30 mA using a Ni-filtered Cu K radiation (λ = 0.15406 nm). The sample was scanned at the ambient condition over scattering 2θ from 10° to 70° with the scanning rate of 0.03°/0.7 s. The crystallinity index (*CrI*) was calculated using Segal’s method (Segal et al. 1959) following the formula:

1 $$CrI = \frac{{I_{200} - I_{AM} }}{{I_{200} }} \times 100\%$$

where I_200_ is the peak intensity at the plane (200) characterized for the crystallinity domain peaked at 2θ ~ 22.8° while I_am_ is the peak intensity at the (101) plane peaked at 2θ ~ 18° characterized for the amorphous domain in cellulose.

Thermogravimetric analysis (TGA, Setaram) was used to analyze the thermal stability and degradation behavior of raw and chemical treated sample. The analysis was performed between ambient and 900 °C under inner gas, with a flow rate of 2.5 L/min at a heating rate of 10 °C/min. The measurement is conducted at the Institute of Chemistry, Vietnam Academy of Science and Technology.

The conductivity of the gel was characterized by measuring the electrochemical impedance spectroscopy (EIS) using an electrochemical workstation (Autolab, USTH, Hanoi, Vietnam). The measurements were performed over a frequency range from 1 × 10⁶ to 10⁻1 Hz with a 10 mV applied voltage. For the measurement, the ionic gel, with a diameter of 16 mm and thicknesses of 1, 3, or 10 mm, was sandwiched between two graphite sheet electrodes. The conductivity is calculated using the following equation:

2 $$\sigma \, = \frac{L}{RA}$$

where L is the thickness of the gel sample, A is the contact area of the gel, and the graphite electrodes and R is the impedance value.

## Results and discussion

**Fig. 1:**
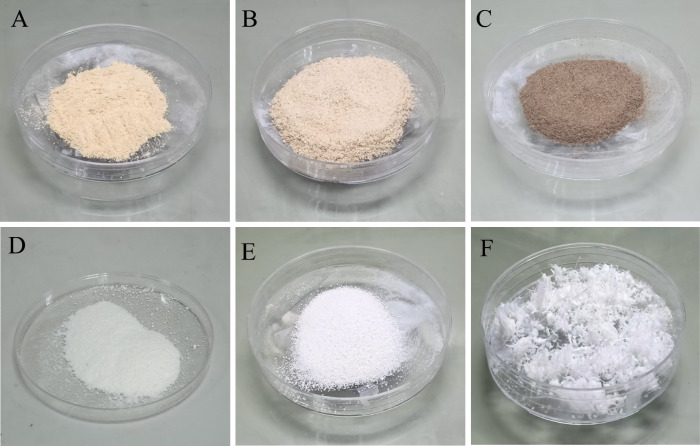
Picture of raw samples of dragon fruit foliage, corn cob and banana pseudostem (**a**–**c**) and their corresponding bleached samples (**d**–**f**)

First, we prepared a PAA solution by adding 10 mL hydrogen peroxide (30%) into acetic acid glacial of equal volumes and then 0.24 mL sulfuric acid 98% as catalyst to accelerate the formation of peracetic acid, the solution was then kept in chemical hood before used. We then mixed the lignocellulose source to liquor with the ratio of 1:10 (g/mL) and conducted the reaction at a temperature of 80 °C for 2 h. After terminated the reaction, we obtained the sample with white color, as shown in Fig. 1 for all the lignocellulosic source. The color transition indicates the removal of chromophore group such as phenolic and ketone in the lignin component, (Zhang and Naebe 2021) which are mainly responsible for the brownish color and light adsorption properties of natural lignocellulosic sources. Our initial result is consistent with several previous reports on using H_2_O_2_ as a delignification method. For examples, Qin et al. (2018) reported the delignification method (NaClO_2_/H_2_O_2_) in which 80% of lignin in woody source was removed with the processing time of 3 h which is much shorter than normal processing time (from 6 to 12 h) (Xia et al. 2021). Li et al. reported a method to remove the lignin chromophore using alkaline H_2_O_2_ hydrothermal solution to reduce the processing time (Li et al. 2017). Xia et al. latter improved the efficiency of the H_2_O_2_ method by incorporating with ultraviolet light illumination, that enable the removal of lignin chromophore occurring with chemical brushing rather than immersion method (Xia et al. 2021).

To diagnose the effect of H_2_O_2_ and acetic acid (glacial) in our solution, we also prepared several solutions including the solution of H_2_O_2_ with 2% (w/w) of H_2_SO_4_ 98% and the glacial acid solution added with 2% (w/w) H_2_SO_4_ 98%. After performing the reaction we observed that the mixture of CH_3_COOH and H_2_SO_4_ did not show the chromophore lignin removal (as shown in Figure S1) while the H_2_O_2_: H_2_SO_4_ needed much longer time (~ 48 h) to partially remove the chromophore lignin which is 24 times longer than the mixture based on peracetic acid. To further diagnose the effect of H_2_SO_4_ concentration, we first prepared the PAA mixture with 0.5% and 5% H_2_SO_4_ concentration, and then perform the bleaching process. We calculated the time to fully bleach the raw samples and we observed that the higher H_2_SO_4_ concentration, the shorter time of the bleaching process, especially for 5% H_2_SO_4_ the processing time is only 1 h as shown in figure S2. The result proves that the processing time of the chromophore removal was in a range of 2–3 h depending on the lignocellulosic source. We noted also that the lignin content in our starting material is usually higher than that of woody source as reported by Xia et al. (2021).

### Chemical components

**Fig. 2:**
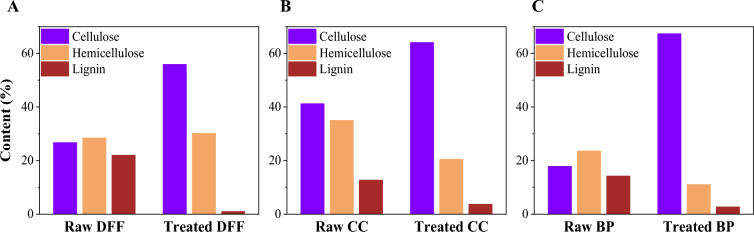
Comparison of cellulose, hemicellulose, and lignin content between raw and bleached samples for **a** dragon fruit foliage, **b** corn cob, and **c** banana pseudostem

To further quantify the chemical components of the sample before and after the chemical treatment, we adapted the procedures reported by the renewable national laboratory with minor modification. Figure 2 presents the content of lignin, hemicellulose, and cellulose before and after chemical treatment and Table 1 summarize all the chemical components. As shown in Fig. 2, the estimated lignin content of the dried agricultural biowaste was 22.5%, 12.74% and 14.27% for DFF, corn cob and banana pseudostem, respectively which is also consistent with other (Fonseca, et al. 2019; Gabriel et al. 2020; Kassim et al. 2014; Stanislas et al. 2022). After the chemical treatment, the lignin content significantly decreased to 1.03, 3.76 and 2.73% for DFF, corn cob and banana pseudostem respectively, the result is also consistent with the observation in color transition after chemical treatment.

**Table 1:**
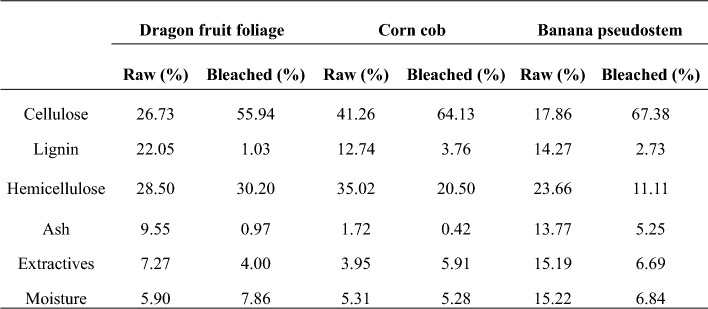
Chemical composition of DFF, CC and BP before and after bleaching

The lignin removal is attributed to the strong oxidizing agents in the liquor including peracetic and hydrogen peroxide that could cleave the β-O-4 linkages, which are prevalent in lignin (Ma et al. 2015). Additionally, sulfuric acid acts as a catalyst for peracetic acid formation and facilitates the breakdown of lignin into smaller fragments, as evidenced by the Klason lignin method. We notice here that the role of H_2_SO_4_ is not only to facilitate the breakdown of lignin components but also the removal of hemicellulose content. In Fig. 2 and Table 1, we also obtained that the hemicellulose content was significantly changed. To quantify the change, we compare the cellulose content to the hemicellulose content, particularly the content ratio of the DFF sample was ~ 0.94 and the ratio of the bleached DFF sample was 1.85, which is approximately two times higher than in the fresh dried DFF. The ratio increase was up to 2.65 and 8.03 for cob corn and banana pseudostem, respectively. The decrease in hemicellulose content indicated that during the chemical treatment, H_2_SO_4_ is not only acted as the catalyst to breakdown the lignin component, but also effectively remove the hemicellulose part. In addition, as reported previously, the acetic acid could behave as a solvent that help to effectively dissolved the lignin component (Zhao and Liu 2013). The notable decrease in ash and extractive content of the bleaching samples also indicates the effective removal of inorganic impurities and non-structural components from the biomass.

Bahloul et al. and others (Bahloul et al. 2021; Song et al. 2019; Risite et al. 2022) reported that for example, to obtain MCC from doum tree, there are three treatment steps including hot distilled water treatment, and then an alkali treatment followed by a bleaching treatment, and importantly both alkali and bleaching treatments should be performed at less three times, (Bahloul et al. 2021) which is convenient method since the lignocellulosic biomass consists of defensive inner structure which has contributed to the hydrolytic stability and structural robustness of the plant cell walls and in addition, the presence of cross-link between cellulose and hemicellulose with lignin via ester and ether linkages leads to the biomass recalcitrance.

Our method allows us to effectively remove lignin, extractive, ash, and the partial degradation of hemicellulose in only one chemical treatment and we attributed the effective of our chemical treatment to the combination effect of removing extractive, hydrolysis the lignin and partially hydrolysis hemicellulose component simultaneously. We also calculated the yield of the chemical treatment by the weight difference before and after the chemical treatment, and we obtained that the reaction yield was 47.5, 60.8 and 40.6% for DFF, CC and BP respectively, which is consistent with the percentage of the cellulose component of the raw materials.

The results demonstrate that our chemical treatment significantly increased the cellulose content in all three biomass samples, while simultaneously significantly remove the content of lignin, hemicellulose, ash, mineral and extractives.

### Morphology and element analysis

**Fig. 3:**
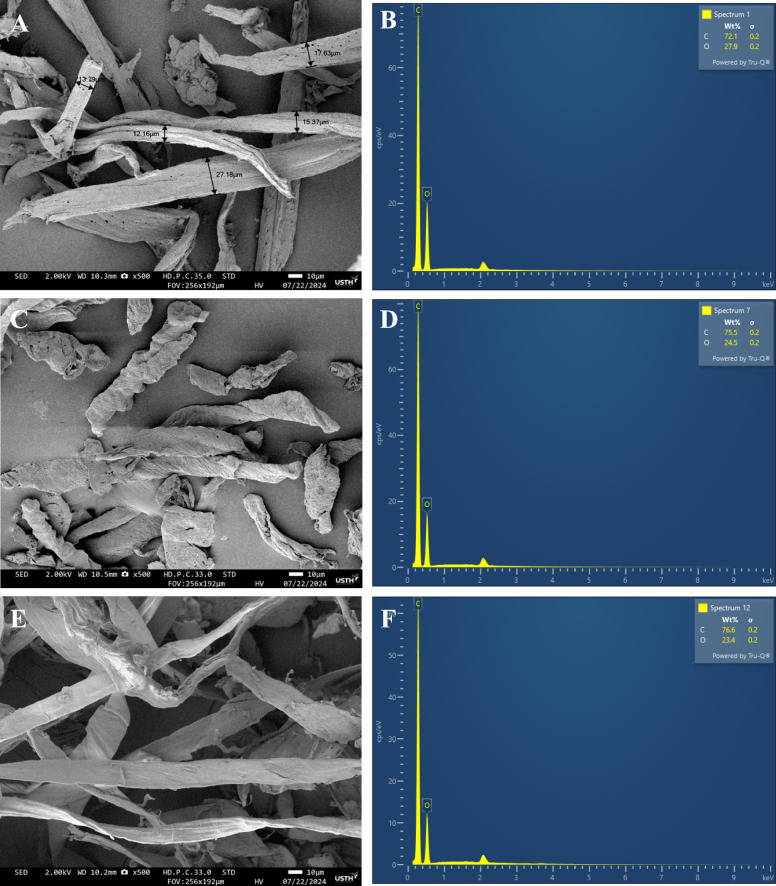
SEM images and EDX spectrums of **a**, **b** dragon fruit foliage; **c**, **d** corn cob; **e**, **f** banana pseudostem after bleaching

The morphologies of the MCC produced using our chemical treatment was shown in Fig. 3. The field emission scanning electron microscopy image (FE-SEM) reveal good homogeneity, smooth surface with the banana pseudostem displaying the smoothest texture among them, and micrometric scale in range of 10–30 μm, which is consistent with other bleached cellulose originating from non-woody sources (Gabriel et al. 2020; Stanislas et al. 2022; Xiang et al. 2017).

The element component of the sample before and after chemical treatment was performed by EDS as shown in Fig. 3, Figure S3 and summarizes in the Table 2. At first, we observed that there are only existence of carbon and oxygen in in all the bleached sample which is significantly different from the raw samples where other elements including K, Na, Ca, Mg, S, Si, P detected by EDS. The element analysis shows the presence of C and O elements with the atomic ratio is identical in comparison to that of the commercial MCC as in Table 2, which proved that our one-step chemical treatment completely removes all mineral component in the fresh dried lignocellulosic source and our MCC has identical chemical composition compared with the commercial MCC.

**Table 2:**
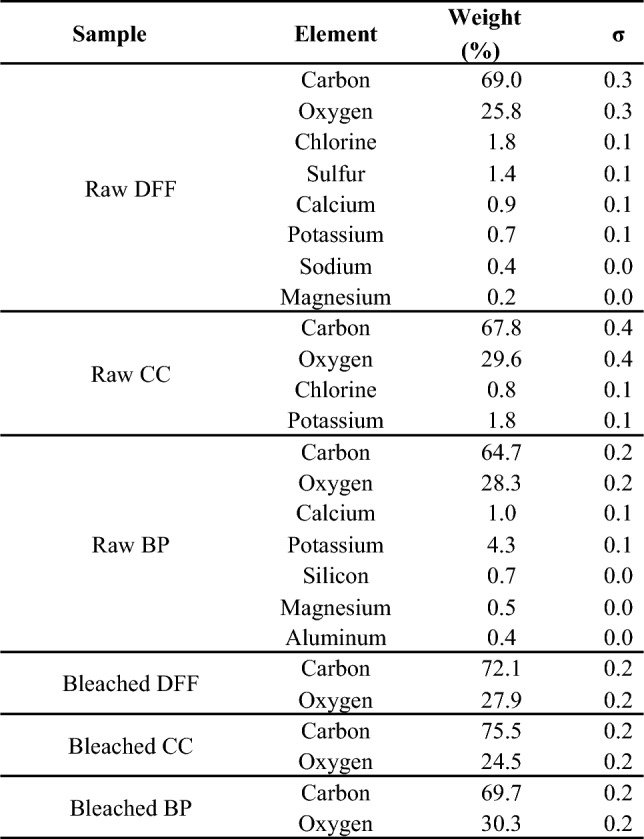
Elemental analysis obtained from EDS for the fresh dried lignocellulosic source and their bleached (MCC) samples

### Chemical structure and crystallinity

**Fig. 4:**
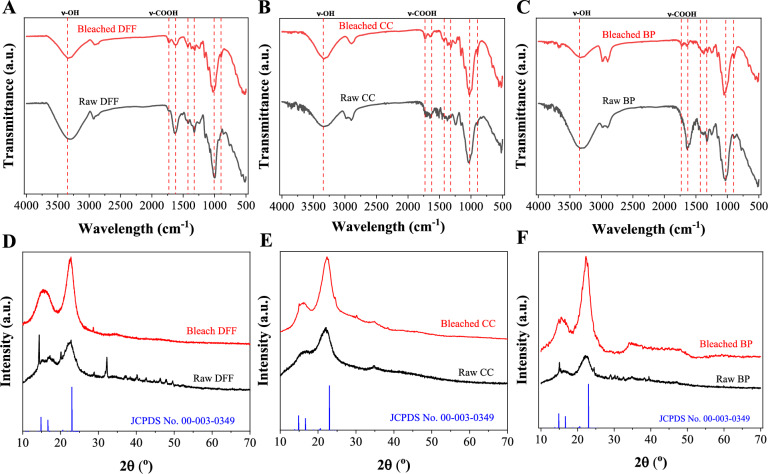
IR spectra of **a** raw and bleached DFF; **b** raw and bleached CC; **c** raw and bleached BP and XRD patterns of **d** raw and bleached DFF; **e** raw and bleached CC; **f** raw and bleached BP. Noted that the bleached sample is the microcrystalline cellulose sample

The chemical structures of the MCC obtained after the chemical treatment are presented and compared with the raw material in Fig. 4a–c. We firstly observed that the FTIR spectra of all bleached samples were almost identical with characteristic peaks at 650, 899, 1020, 1320, and 1427 cm⁻^1^ as an indicative of cellulose component (Xu et al. 2013). In the spectra of raw samples, there are vibrational bands at 1514 and 1460 cm^−1^ due to C = C and C = C–C groups stretching vibration of lignin component; however, these bands almost disappeared in the spectra of the bleached cellulose. Additionally, a new peak emerged at 1720 cm⁻^1^, corresponding to carboxylic acid groups and the vibrational band peaked at 1242 cm^−1^, assigned to the symmetrical vibration of the COO^−^ group, which could be explained by the existence of the -COOH group functionalized on microcrystalline cellulose. Interestingly, the vibrational band peaked at 1720 cm^−1^ in the raw DFF sample is usually assigned to the vibration of the C = O chemical group in lignin, however, the vibration still existed in the bleached DFF which could be generated during the bleaching process using oxidative chemical compounds. In summary, the FT-IR data indicated the removal of lignin and hemicellulose and the introduction of -COOH group on microcrystalline cellulose, while the structure of cellulose remained unchanged.

The XRD patterns for both raw and bleached samples are presented in Fig. 4d–f. All spectra display three prominent peaks at 2θ approximately 15.55°, 22.56° and 34° which is the signature of the cellulose I structure as expected for natural cellulosic material. We observed that the intensity of these peaks in bleached sample is greater than that of raw sample, which suggests the removal of most amorphous regime and non-cellulosic components, lignin, and hemicellulose. As presented, there are several peak at large angle 2θ in the raw samples which is the indication of mineral inorganic component, and it was disappeared on in the bleached samples which is consistent with elemental analysis. Based on the data and Eq. (1), the crystallinity indices (*CrI*) for the raw and bleached DFF samples are calculated to be 43.3 and 61.3%, respectively; for the CC samples, the indices are 37.8 and 50.1%; and for the BP samples, they are 51.2 and 75.2%. To summary, the XRD result indicated that our one-step chemical treatment shown: firstly (i) the effective removal of these impurities, Lignin, and hemicellulose components and secondly (ii) the effective dissolution of the amorphous regions of cellulose which ultimately enhances the overall crystallinity of the bleached samples.

### Thermal stability

**Fig. 5:**
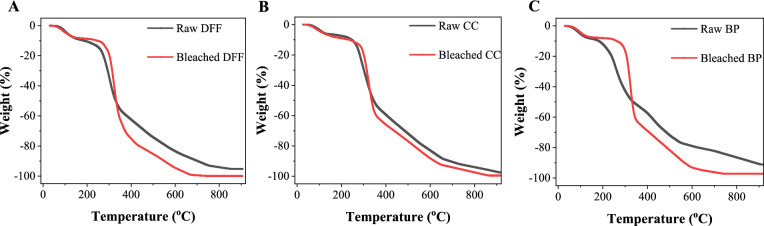
TGA curves of **a** raw and bleached DFF; **b** raw and bleached CC; **c** raw and bleached BP

The thermogravimetric analysis of both raw and bleached samples is presented in Fig. 5. It is known that there are 3 steps of lignocellulosic degradation (Bridgwater 2012). The initial step of degradation is in the temperature range of 0–200 °C at T_onset_ ~ 110 °C, which could be attributed to the loss of moisture with a weight loss of approximately 10%. The second step is the degradation from ~ 200 °C to 600 °C where the crystalline region and amorphous was started to decompose with a significant weight loss (70–80%) due to the gas phase transition and tar formation. In this stage, the T_onset_ of all bleached sample kept similar at 270 °C and is slightly higher than that of the raw samples, which is reasonable due to the presence of volatile compound in the raw samples. In the last stage in the temperature range of 600 °C–800 °C, the decomposition of the material is complete to form volatiles and tar. We also note that the tar concentration in the raw sample is significantly greater than that of the bleached sample, for example the tar concentration of the bleached DFF is only approximately 4% which is 3 times lower than that of the raw DFF sample. The result proven the removal of mineral and lignin components after the chemical treatment.

### Cu-cellulose gel electrolyte formation

**Fig. 6:**
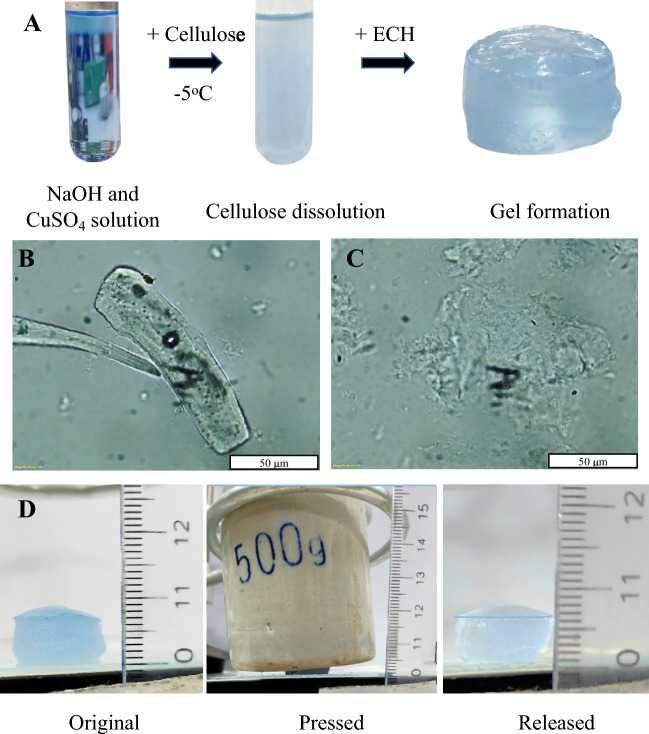
**a** Step by step formation of cellulose GPE: First, preparation of NaOH and CuSO_4_ solution; then addition of cellulose and cooled the mixture down to − 5 °C for 1 h and finally adding ECH for gel formation. Optical microscopy of **b** bleached DFF and **c** dissolution of bleached DFF. **d** Shape of Cellulose hydrogel before and after under stress of object with 500 g

Next, we attempted to make an electrolyte gel based on our MCC by directly dissolve microcrystalline cellulose in NaOH solution and then gelation by adding ECh compounds as illustrated in Fig. 6a. At first, the dissolution of cellulose was done by both in NaOH solution and the mixture of Cu(OH)_4_^2−^ and NaOH solution at low temperature (~ − 5 °C). The dissolution of cellulose in NaOH was reported previously (Budtova and Navard 2016; Liebert 2010) and is facilitated by several mechanisms such as NaOH disrupts the hydrogen bonds within the cellulose chains, increasing their flexibility and promoting partial hydrolysis of the glycosidic bonds. The resulting cellulose chains interact with Na⁺ ions to form alkali cellulose, which enhances solubility (Liebert 2010). In addition, we also prepared a mixture of Cu(OH)_4_^2−^ and NaOH by dropwise the CuSO_4_ solution into 6.25 M NaOH solution as several report shown that the Copper ions in form Cu(OH)_4_^2−^ can form coordination bonds with celluloses, which then may increase the solubility of cellulose (Ogawa et al. 2014b; Saalwächter et al. 2000; Saalwächter and Burchard 2001). We observed that both solutions can dissolve our MCC due to the color transition from the opaque with visible cellulose clusters into a translucent solution devoid of cellulose clusters after undergoing a dissolution process at − 5 °C in 1 h. We performed the observation on both solution before and after dissolution process by optical microscopy as in Fig. 6b, c, and we observed that after the dissolution process, there are neglectable microcrystalline cellulose particles observed and generally the width and length of the observed particles is much smaller than that without dissolution process.

In the final step of gel formation, epichlorohydrin (ECH) was introduced into the mixture, forming ether linkages with cellulose, and establishing a stable three-dimensional structure that supports gel formation and the Cu-cell GPE is illustrated in Fig. 6a. The mechanical properties of the gel are illustrated in Fig. 6d, demonstrating that a gel with thickness of less than 1 cm and a diameter of 1.6 cm can support a mass of 500 g, corresponding to 25 kPa, without changing the gel shape and, the gel additionally exhibits elasticity, as it reforms after the mass was removed.

### Ionic conductivity of Cu-cellulose gel electrolyte

**Fig. 7:**
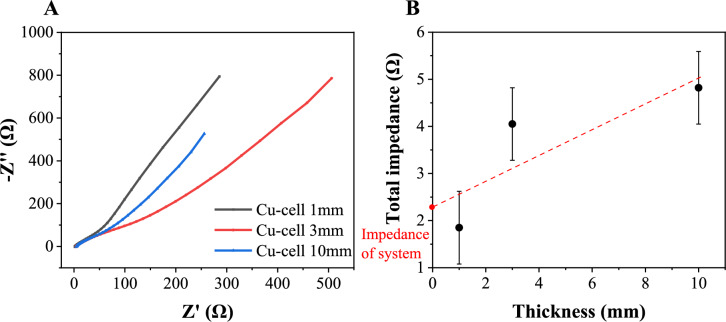
**a** EIS curves of Cu-cell gel at different thickness and **b** total impedance of the gel at different thickness, each measurement was performed at least 3 times

The electrochemical impedance spectroscopy (EIS) Nyquist plots of the GPEs measured at room temperature with varied thickness in Fig. 7a and total impedance to thickness in Fig. 7b. All Nyquist plots exhibited a linear trend without a semicircle, indicating that the ionic conduction within these GPEs is a non-Faradaic process (Ye et al. 2020). Therefore, the total impedance of the hydrogel is the intercept of the plot and the real axis, giving the impedance value of all the shown gel in Fig. 7b. From there, the impedance of each gel is calculated by subtracting the total impedance by the impedance of the system, then with the provided data and Eq. (2), the conductivity of gel 1 mm, 3 mm and 10 mm is 110.5, 85.2 and 197.3 mS/cm. Table 3 summarize the conductivity of hydrogel made of cellulose by other and in comparison, with our ionic gel. As shown, our hydrogel shown much greater conductivity compared to others and closer to the value reported by Chunpeng Yang et al. (Yang et al. 2021) and Qi Dong et al. (~ 0.2 S. cm^−1^), (Dong et al. 2022) which could also be attributed to the effect of the coordination of copper ion (Cu^2+^) within cellulose chain (Yang et al. 2021; Dong et al. 2022).

**Table 3:**
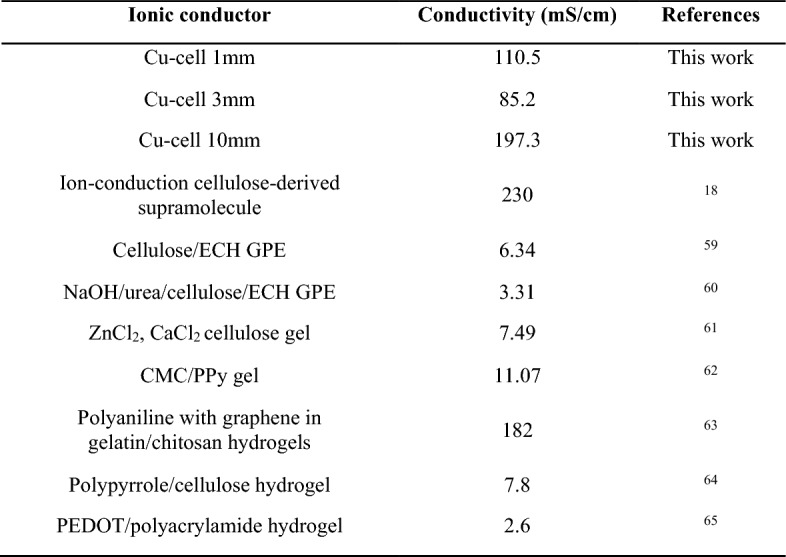
The conductivity of Cu-cell gel compares with gel in several related studies

## Conclusion

In this study, we demonstrate an effective, single-step method for extracting microcrystalline cellulose with a width of 10–50 μm and length of 100–1000 μm from various agricultural waste sources, including dragon fruit foliage, corn cob, and banana pseudostem, using a PAA solution added with 2% H₂SO₄. Key findings reveal significant changes in chemical composition, with cellulose percentage increasing substantially across all biomass sources after bleaching: dragon fruit foliage increased from 26.73 to 55.94%, corn cob from 41.26 to 64.13%, and banana pseudostem from 17.86 to 67.38%. In addition, delignification and the removal of hemicellulose and mineral was performed effectively, for example the substantial reduction in lignin content: dragon fruit foliage decreased from 22.05 to 1.03%, corn cob from 12.74 to 3.76%, and banana pseudostem from 14.27 to 2.73%. XRD confirmed that the crystallinity of the bleached cellulose is much greater than the raw sample, and elemental analysis shown the existence of O and C elements in the bleached sample. We attributed the effective of our chemical treatment to the combination effect of removing extractive, hydrolysis the lignin and partially hydrolysis hemicellulose component simultaneously. Our microcrystalline cellulose obtained can serves as an effective matrix for gel polymer electrolytes, demonstrating promising mechanical and electrical properties with highest ionic conductivity of 197 mS/cm. Our study opens a new, simple path to directly extract microcrystalline cellulose from lignocellulosic source and extend the application of microcrystalline cellulose toward applications in energy storage and conversion.

## Supplementary Information

Additional file1 (DOCX 6436 kb)

## Data Availability

The datasets used and analyzed during the current study are available from the corresponding author on reasonable request.

## Abbreviations

ATR-FTIR: Attenuated total reflectance Fourier-transform infrared spectroscopy
MCC: Microcrystalline cellulose
XRD: X ray diffraction
FE-SEM: Field emission scanning electron microscopy
EDS: Energy dispersive X-ray spectroscopy
TGA: Thermogravimetric analysis
DES: Deep eutectic solvent
DFF: Fine-dried Dragon Fruit foliage
CC: Corn cob
BP: Banana pseudostem
ECH: Epichlorohydrin
