# Supplementary material for: A one-step chemical treatment to directly isolate microcrystalline cellulose from lignocellulose source
Source: Bioresour Bioprocess. 2025 Aug 22;12(1):89. doi: 10.1186/s40643-025-00920-6 (PMC12373626; doi:10.1186/s40643-025-00920-6)
Supplement: Supplementary file 1 — Additional file1 (DOCX 6436 kb) [file 40643_2025_920_MOESM1_ESM.docx]

**Supplement Information**

A One-Step Chemical Treatment to Directly Isolate Microcrystalline Cellulose from Lignocellulose Source

Thai Anh Do,^1, #,^ Van Quyen Nguyen,^1, #, *^  Thi Minh Chau Nguyen,^2^ Thi Hang Nga Nguyen^3^, Thi Huong Le^1^

^1^Department of Advanced Materials Science and Nanotechnology, University of Science and Technology of Hanoi, Vietnam Academy of Science and Technology, 18 Hoang Quoc Viet, Cau Giay 11307, Hanoi, Vietnam.

^2^ Faculty of Chemistry, University of Science, Vietnam National University, 19 Le Thanh Tong, Hoan Kiem, Hanoi, Vietnam.

^3^ Faculty of Mechanical Engineering, Thuy loi University, 175 Tay Son, Dong Da, Hanoi, Vietnam.

*# Equally contribution.*

** Corresponding author email:* [*nguyen-van.quyen@usth.edu.vn*](mailto:nguyen-van.quyen@usth.edu.vn)

KEYWORDS: Lignocellulosic source, Microcrystalline Cellulose, Peracetic acid, Ionic cellulose conductor.

**Figure S1:** Picture of the dragon fruit foliage samples after reaction with the solution of (A) CH_3_COOH: H_2_SO_4_ and (B) H_2_O_2_:H_2_SO_4_.


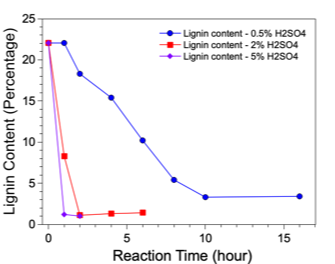


**Figure S2:** the plot present the dependence of lignin content and the reaction time between the raw DFF and PAA-0.5%H2SO4 (blue curve), PAA-2%H2SO4 (red curve) and PAA-5%H2SO4 (violet curve).

**S1. Scanning electron microscopy and EDS analysis**

Field Emission Scanning Electron Microscopy (FE-SEM). The microcellulose solution was spin coated on SiO2 (100) wafer, and then the wafer sample was dried at 40^o^C under vacuum overnight. The sample was then coated with 15 nm of Ti by e-beam evaporator (lesker PVD-75) at vacuum 10^-6^ torr and depositing rate 0.2 nm. s^-1^. The sample was then imaged by FE-SEM (JEOL, JSM-IT800) at few kV. EDS mode was conditioned by using an accelerating voltage of 20 kV, a working distance of 10 mm, and the accumulation time of 1 minute.

**Figure** **S3**. SEM with low magnification and EDS of DFF (A and B), Cob corn (C and D), BP (E and F) and commercial MCC (G and H). The EDS data (B, D, F) showed the mineral elements existed in the raw materials.
